# A qualitative cross-cultural analysis of NICU care culture and infant feeding in Finland and the U.S.

**DOI:** 10.1186/s12884-019-2505-2

**Published:** 2019-10-10

**Authors:** Sarah Holdren, Cynthia Fair, Liisa Lehtonen

**Affiliations:** 1 Department of Pediatrics, Turku University Hospital, Turku, Finland; 2 Public Health Studies & Department Chair, Elon University Department of Public Health Studies, Elon, North Carolina USA; 3 Department of Pediatrics, Neonatology & Professor of Pediatrics, Turku University Hospital, Turku, Finland

**Keywords:** Infant feeding, Breastfeeding, NICU, Care culture, Parent-infant closeness, Family-centered care, Family-integrated care

## Abstract

**Background:**

The benefits of family-centered care for the health and well-being of preterm infants and their families include increased parent-infant closeness, improved lactation, and positive mental health outcomes; however, it is known that the extent to which family-centered care is adopted varies by unit. This study aimed to understand how differences in neonatal care culture in two units in Finland and the U.S. were translated to parents’ infant feeding experiences in the hope of improving relationally focused feeding practices in both locations.

**Methods:**

This qualitative, cross-sectional study utilized narrative methodologies to understand the lived experiences of 15 families hospitalized in a tertiary neonatal intensive care unit in Finland (*n* = 8) and the U. S (*n* = 7).

**Results:**

A global theme of *lactation as a means or an end* showed that lactation and infant feeding were framed differently in each location. The three supporting themes that explain families’ perceptions of their transition to parenthood, support as a family unit, and experience with lactation include: universal early postnatal challenges; culture and space-dependent nursing support; and controlled or empowering breastfeeding experiences.

**Conclusions:**

Care culture plays a large role in framing all infant caring activities, including lactation and infant feeding. This study found that in the unit in Finland, breastfeeding was one method to achieve closeness with an infant, while in the unit in the U.S., pumping was only an end to promote infant nutritional health. Therefore, breastfeeding coupled with closeness was found to be supportive of a salutogenic, or health-promoting, care approach for the whole family.

## Background

The neonatal intensive care unit (NICU) has previously been characterized by restrictive care cultures that limit parental access to the infant, with some parental limitations still existing in many places even today [1]. Within the past decade, a turn toward family-centered care (FCC) has expanded the role of the parent in the NICU and has become the standard of practice recommended by the American Academy of Pediatrics [2–4]. Attributes of FCC vary from unit to unit; however, scholars and clinicians generally agree that this type of care is characterized by unlimited parental presence, shared responsibility of the infant’s hospital care, and open communication between parents and the NICU care team [5, 6]. This care approach has shown ample benefits for the health, well-being, and overall satisfaction with care for infants and their families, as well as the possibility to decrease length of stay in the NICU [7, 8].

Because parental presence serves as the foundation of FCC, it is unsurprising that this approach to care also has positive benefits for infant feeding. An exclusively human milk based diet is the recommended nutrition for preterm infants, especially those born very-low-birth-weight (VLBW), as this diet has been shown to increase feeding tolerance, improve neurodevelopment, and decrease risk of severe infection, sepsis, or gastrointestinal disease [2, 9]. While there are evident barriers to providing this type of diet to neonates, including difficulties with lactation initiation due to preterm birth and increased maternal stress [10], FCC that decreases parent-infant separation can help alleviate some of the challenges associated with expressing human milk in the NICU [11, 12]. Because mothers of preterm infants often require skilled lactation support to have success with lactation, lactation-supportive care cultures that prioritize both the biological and psychological aspects of lactation may have positive impacts for the long term health and well-being of the mother-infant dyad even after NICU discharge [11, 13, 14].

While FCC may make providing an exclusively human milk based diet to neonates more feasible, certain care cultures may be more breastfeeding friendly than others [15]. Preterm infants are often unable to initially feed from breast, and therefore, mothers require additional support to transition their infant to breast both in the hospital and at home [16–18]. Many NICUs still have policies that postpone any breastfeeding attempts until a certain gestational age; however, this may cause the infant to miss the developmental window to begin eating from breast [19, 20]. Furthermore, the benefits of direct breastfeeding to the mother should not be overlooked, especially as related to establishing a relational bond with the infant [21–23].

Similar to the variation in care cultures surrounding breastfeeding, some studies have found that FCC’s translation to the parent experience varies [6, 24–26]. Barriers still exist that limit parental presence in many NICUs. This includes limited access to single-family rooms or other amenities necessary for families to stay comfortably in the NICU [27, 28]. Single-family rooms have garnered ample scholarly attention [29–31] with many studies suggesting that it is the family-staff interactions and parental autonomy supported by these environments, and not necessarily just the architecture, that may make them successful [32–34]. Furthermore, the ways in which parental autonomy is supported in these environments has been shown to result in “attuned feeding,” or feeding that is relationally aware and supportive of a healthy mother-infant dyad [35]. For this reason, it is evident that the extent to which FCC is adopted can directly impact the infant feeding experiences of families in the NICU.

Nordic countries have shown success with the adoption of FCC and are advanced in their utilization of human milk in the NICU [1, 36–38]. There is speculation that part of this success is due to the long parental leaves and generous social welfare policies present in these nations [39–41]. Parental leave is of particular importance when considering parental presence in the NICU, and has been cited as one of the challenges associated with the adoption of single-family rooms in the U.S., especially for parents of lower socioeconomic status [29, 42, 43]. Conversely, current work in Nordic NICUs is focused on not only having parents present in the NICU, but also on having “zero separation” between parents and infants from the first moment after birth until hospital discharge via couplet care [44, 45]. Of additional importance to the success of FCC in Nordic countries is their medico-legal philosophy, as the responsibility of care for a hospitalized infant may be more fully transferred to the family with limited legal risk in these national contexts in comparison to the U.S. [46].

Narrative methods are useful to understand the lived experiences of families within different healthcare environments [47, 48]. Ample attention has been given to the experiences of families in the NICU, both regarding FCC and infant feeding, but most of these studies are single-sited and do not compare narratives across care cultures [49–52]. While some qualitative studies have compared different NICU settings [35, 53, 54], there are no currently published qualitative studies addressing parents’ perspectives on care culture and its impact on infant feeding in these two unique neonatal settings in the United States and Finland. The purpose of this study was to understand how the differences in care culture between two tertiary NICUs in the U.S. and Finland are translated to the infant feeding experiences of families in order to better understand how to improve relationally focused feeding practices in both locations.

## Methods

### Design

This cross-sectional, qualitative study aimed to understand the lived experiences of mothers who decided to breastfeed or pump milk for their very preterm (< 32 weeks’ gestation) neonate in two different neonatal intensive care settings. The U.S. data include narratives from a single site that were gathered as part of a larger multi-sited ethnographic research study on human milk feeding in U.S. neonatal intensive care settings. Data from Finland are gathered from families hospitalized in a single, regional unit using the same interview methodology as in the U.S. Ethics approval to conduct this study was granted by the Elon University Institutional Review Board and the Research Ethics Board of the Hospital District of Southwest Finland.

### Setting

Participant observation was completed in both settings to gain a broad understanding of the unique care cultures in each unit. The U.S. unit is a regional tertiary care unit housed within a children’s hospital. It is not attached to a birthing hospital, but receives infants via transfer from birthing hospitals throughout the region. Most infants in this unit are placed in an open-bay NICU architecture. The Finnish unit is a regional tertiary care unit housed within a children’s and women’s hospital, meaning that nearly all infants admitted to the unit are birthed in the same hospital. Most infants in this unit are placed in a single-family room for the majority of their stay. The Finnish unit has access to an on-site milk bank, while the U.S. hospital receives its milk from an external milk bank. The catchment area for the Finnish unit includes three level two hospitals, one of which is located on an island. The furthest drive for families of hospitalized infants is approximately 6 h by ground transportation. The families coming from the small island must take an overnight ferry to reach the hospital. The U.S. unit is an all-referral unit, with most families residing within approximately a 5-h drive to the hospital, but with some coming from elsewhere in the continental United States. However, it is important to note the differences in healthcare organization between these two countries, as hospital access is solely based on location in Finland, while location and accepted medical insurances play a role in hospital access in the U.S.

Both units encourage parents to be present for medical rounds, but take a different philosophical approach in their inclusion in medical decision-making [24, 25]. Parent participation in infant care is generally more encouraged in the Finnish unit than in the U.S. unit, although both units support families to practice skin-to-skin with their infants early on in their NICU stay [1, 55]. Neither unit has the WHO Baby-Friendly Hospital Initiative (BFHI) certification, but both are well-known for their advances in FCC within their national contexts. Therefore, neither of these units represents the typical neonatal experience within the U.S. or Finland, but serve as exemplars of FCC in these two countries. More detailed descriptions of each unit’s supportive amenities for families are provided in Table 1.

**Table 1:** Descriptions of Supportive Accommodations for Families

Accommodation	Finnish Unit	U.S. Unit
Family Visiting	Parents, including siblings, may stay overnight at the bedside as long as siblings are healthy. Other family members are welcomed to visit as desired per parent consent.	Parents, but not siblings or other family members, are invited to stay overnight at the bedside. Other healthy family members can visit during daytime hours per parent consent.
Sleeping Arrangements	One or two adult hospital beds are provided based on space in the infant’s room. In addition, sleeping rooms are available within the hospital. Families from out of town are also provided an apartment near the hospital to stay if desired.	A recliner at the bedside is provided. There is limited access to sleeping rooms away from the bedside, which are usually used for families of infants close to discharge. Families from out of town are invited to stay in the Ronald McDonald House on the hospital campus.
Bathroom Arrangements	50% of single family rooms have a private bathroom with shower. The rest may access the bathroom and shower a short walk down the hall.	There are no private bathrooms or showers provided in the unit, but parents who stay overnight may access showers in a separate part of the hospital.
Kitchen Access	A full kitchen is shared among families on the unit and may be used to store and cook food.	A kitchenette is accessible in the common area just outside the unit.
Laundry Access	Laundry machines are located on the unit and accessible to all families.	There is no laundry access for families.
Common Areas	Two common areas are provided for families on the unit: one living room where social events and family classes are held, and one coffee/dining room where meals can be eaten.	A common area is located just outside the unit with a kitchenette, couches, and dining tables.
Pumping and Milk Storage	Mothers pump at the bedside with either the single-family room door closed or a curtain drawn for privacy if desired. Fresh milk is stored in a small fridge at the bedside and families have access to the milk kitchen where milk for fortification or already fortified milk is placed.	Mothers may pump at the bedside or in a pumping room on the unit. If pumping at the bedside, a curtain may be drawn for privacy. All milk is stored in the milk kitchen and nurses manage the milk storage and access process.

### Participants

Written and oral information was provided to both sets of participants before informed consent. U.S. participants were recruited via a hospital family advocate, who reached out to recently hospitalized, eligible families and shared study information between August 2016–June 2017. Finnish participants were recruited by S.H. and a Finnish interpreter during the last month of the eligible family’s hospitalization between October 2018–May 2019. The inclusion criteria were that the infant had been born very preterm (< 32 weeks’ gestation) and VLBW (< 1500 g), and that the mother had intended to breastfeed and/or pump milk at the time of birth. Some families in Finland were excluded if they were not proficient in English. Using a grounded theory approach, study recruitment during each recruitment period ended once thematic saturation in the participants’ narratives was reached [47, 56].

A total of 15 families participated in this study, with 7 from the U.S. unit and 8 from the Finnish unit. Each set of participants included a total of 11 infants. The average gestational age of the infants was 27 weeks and there were 3 sets of multiples (2 sets of twins and 1 set of triplets in the U.S., 3 sets of twins in Finland) in each set of participants. Average maternal age at birth was slightly lower in the Finnish participants (29 years versus 31 years) and more U.S. mothers were primiparous. Relevant demographic information can be found in Table 2.

**Table 2:** Participant and Infant Demographic Information

Characteristic	U.S (%)	Finland (%)
Gestational Age		
23–27	7 (63.6)	7 (63.6)
28–32	4 (36.4)	4 (36.4)
Maternal Age at Birth		
20–24	1 (14.3)	0 (0)
25–29	2 (28.5)	3 (37.5)
30–34	3 (42.8)	1 (12.5)
35–39	0 (0)	3 (37.5)
40–44`	1 (14.3)	1 (12.5)
Multiples		
Twins	2 (18.2)	3 (27.2)
Triplets	1 (0.10)	0 (0)
Parity		
Priamparous	5 (71.5)	3 (37.5)
Multiparous	2 (28.5)	4 (50.0)
Grand Multiparous	0 (0)	1 (12.5)
Birth Method		
Vaginal	3 (42.8)	5 (62.5)
C-Section	4 (57.1)	3 (37.5)
Education		
High School	2 (28.5)	3 (37.5)
Two or Four Year College	3 (42.8)	4 (50.0)
Graduate	2 (28.5)	1 (12.5)

*The U.S. sample contains 7 families and a total of 11 infants. The Finnish sample contains 8 families and a total of 11 infants

### Data collection

A series of narrative interview prompts were initially designed to elicit participants’ stories regarding infant feeding and lactation in the NICU [47]. Prompts and elicitation techniques were first piloted with a small number of volunteers in the U.S., who helped refine the final series of questions used for data collection [56]. The same interview techniques were utilized in Finland, and the interpreter and interviewer piloted these questions in the Finnish setting to ensure that they were interpreted by the participants properly and that probing would occur when necessary. Examples of interview prompts are provided in Table 3.

**Table 3:** Example Interview Prompts

Topic	Prompts
Becoming a Parent in the NICU	Tell me your NICU story. What expectations did you have for your pregnancy and birth? Describe the first time you saw your infant. How did/does being in the NICU make you feel? Describe your day to day NICU routine.
Infant Feeding	What were your infant feeding intentions? How was/is your infant fed while in the NICU? Describe the infant feeding education and support you experienced. What was the first time you pumped, did skin/skin, breastfeeding, etc. like?
Provider Interactions	Describe a time you discussed infant feeding with a healthcare provider. How did the NICU staff support you in infant feeding? Describe a time you negotiated with a provider about your infant’s care.
Parenting Post Discharge	How has the NICU impacted your parenting today? What are your hopes for your child’s nutrition in the future?

U.S. participants were interviewed either in-person (1) or by telephone (6), based on their preference. Finnish participants were interviewed in the participant’s single-family room. All U.S. interviews were conducted in English, and all Finland interviews were conducted in English with the assistance of a Finnish interpreter who translated medical terminology and figures of speech. Basic demographic information was collected after the interview.

### Analysis

All interviews were audio recorded, transcribed verbatim, and imported into Dedoose (Dedoose V6.1.18) for analysis. Quality and completeness of the U.S. transcriptions were confirmed by S.H. and C.F.. Quality and completeness of the Finnish transcripts were confirmed by S.H. and the Finnish interpreter.

Thematic narrative analysis was used to analyze participants’ interview responses [47]. Themes emerged from the data via line-by-line coding of the text and constant comparison across the data sets [56]. The U.S. and Finland data were first analyzed separately. For each set of data, analysis began with the authors (S.H. and C.F. in the U.S., S.H. and L. L in Finland) familiarizing themselves with the data. Line-by-line coding was led by S.H. for both sets of data and was confirmed during frequent discussions with the authors (C.F. in the U.S. and L.L. in Finland). After each set of data had been analyzed, all authors discussed the similarities and differences between the two sets of data and highlighted relevant areas of thematic saturation between both. The global themes and subthemes that arose from the comparative analysis of both sets of data are reported below.

## Results

**Fig. 1:**
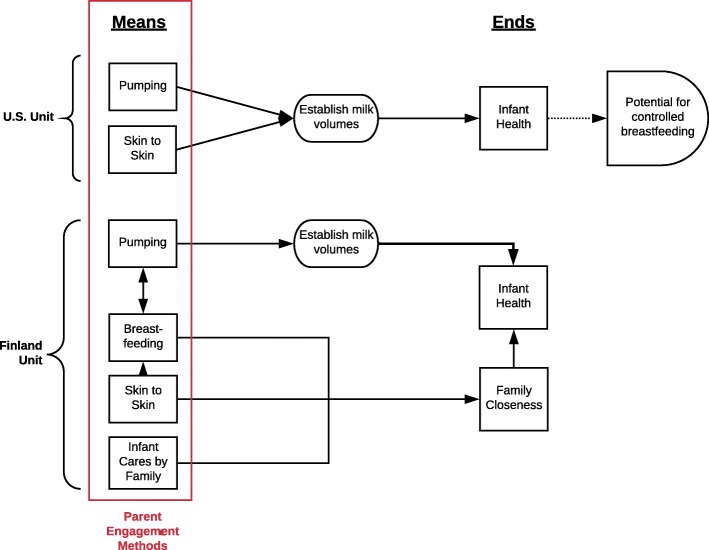
Conceptualization of the Global Theme, “Lactation as a Means or an End”. This diagram outlines the different ways parents are allowed to interact with their infants (“means”) and the clinical intention for these engagement methods (“ends”). This diagram further elucidates how breastfeeding is one mean to achieve closeness in Finland, while pumping is one of few means for parents to contribute to infant health in the U.S

A global theme of *lactation as a “means” or an “end”* emerged from the data set and is conceptualized in Fig. 1. This theme relates to how lactation is framed by the staff and embodied by the families in each unit based on the implementation of FCC. The three supporting themes that explain mothers’ perceptions of their transition to parenthood within the NICU, their overall support as a family unit, and their experience with lactation include: universal early postnatal challenges; culture and space dependent nursing support; and controlled or empowering breastfeeding experiences. These themes underpin the concept that in the unit in Finland, direct breastfeeding is a “means” for closeness, while in the unit in the U.S., pumping serves as an “end” for health.

### Universal early postnatal challenges

Participants in both settings described their initial difficulty immediately after preterm birth, suggesting that a birth that was different than expected led to struggles with maintaining a sense of time and transitioning to parenthood. It was also evident in these narratives that separation or closeness immediately after birth impacted the family’s ability to cope with the initial stresses of the NICU and begin taking on the role of a parent.

When describing preterm birth, many participants used words such as “shock” and “worrisome” to characterize the unexpected nature of being thrust into the postnatal period. One participant in the U.S. stated: *“umm, I remember feeling her move, and like really start to move that one week and then that’s all I had. It was over in the blink of an eye” (US5).* For many, a normal pregnancy had preceded an unexpected preterm birth, making it feel extremely expedited. One Finnish participant discussed her normal pregnancy up until it was discovered in the second trimester that her pregnancy was ectopic and stated: *“Everything just came. We just expected that everything was fine, so it all hit like a wall…So it was … a little bit stressful” (F6).* Feelings of being blindsided were common among both sets of participants even when preterm birth was expected, as this U.S. participant describes: *“Umm, there were no warning signs. I was classified high risk due to some blood issues I had a couple years ago, but I was low risk on the high risk scale. So nothing should’ve gone wrong, umm, but for whatever reason I went into labor” (US4).*

Compounding these feelings of stress at preterm birth, participants described a “jumbled up” sense of time and a struggle to feel close with their infant. One Finnish participant describes the ways that they were overwhelmed with information immediately after birth: *“We lost the sense of time. Yeah it was because we were like, like in shock after the c-section and the whole situation. It took like maybe two weeks that we were in a state of you know … stress …*” *(F3).* Some participants attributed this confusion to separation from their infants. A Finnish participant stated: *“I think because the beginning was so, everything went so quickly … and I couldn’t hold them or see them so, I didn’t get the connection at all. Making the connection took time” (F2).* This feeling was exaggerated in the U.S. participants, as few were able to stay comfortably overnight in the unit. One participant describes the first night leaving her infant in the NICU after her partner prompted her to return home and rest in bed instead of in the chair provided at her infant’s bedside: *“I was adamant I was not leaving. I was like ‘You’re taking my heart out of my chest, I, I can’t … making me leave her behind, how am I supposed to breathe?’” (US5).*

Parent-infant separation, although imposed to a different extent in each unit, impacted participants’ perceptions of themselves as parents. One Finnish participant describes how she had to remind herself that she became a mother: “*At home, I actually would forget that I even had babies and I would have these pictures like reminding me … like yeah you have babies. You are a mother*” (F1). A mother in the U.S. described having a similar problem, but at the bedside. She stated: *“You know, it was hard initially for me to just like adjust to knowing that this little human was mine” (US3).* Participants described eventually claiming their roles as parents, but some were unsure what caused them to do so: “*Now I feel like a parent, but it took time for me. In the beginning if somebody said to me like ‘Hey mom,’ I would be like ‘Huh, no’ … But now it changed. I don’t know what changed it. Just something changed after a couple of weeks” (F1).* Others in Finland argued that it was the enhanced closeness that occurred once the infant was allowed to be in kangaroo care or was moved to an open bed instead of an incubator that made the difference:

*“At first I think … the idea was is this really my baby? Especially in the isolette. And then when he changed to this bed he came much closer and then I think we both started to do much better … You always see better the changes in his face and the feelings and then I think it is easier if you know the baby better” (F8).*

For this reason, some participants reflected on the first few days to weeks of their NICU stay as the most difficult, even suggesting that it was the *“lowest point of [the] stay” (US3).*

### The role of culture and space dependent nursing support

Many mothers described nursing support as an integral part of their NICU experience, especially as related to lactation and infant care. It is evident from the participants’ narratives that both NICU space and care culture impact perceptions of nursing support in each unit. In both infant care and lactation, participants in the U.S. suggested that the nursing approach in the open-bay unit produced barriers that caused them to feel a sense of failure, while participants in Finland described the ways that the nursing approach within the single-family room helped them take care of both their infant and themselves.

Participants in Finland described feeling a sense of autonomy in their single-family room, suggesting that nurses gave them the space to process their emotions and begin to get to know their infant:

*“It kinda felt like you can do … what you want more freely...because things like, like talking to the baby or singing to the baby … even though it kinda feels like the most normal thing, but when you have someone else in the room you kind of feel a bit more self-conscious. And bursting into tears uhh next to someone who you don’t know … it’s not like the most, the most, most comfortable thing” (F2).*

As parents slowly got to know their infant, nurses taught and encouraged them to begin doing more of their infant’s daily cares. One participant stated:

*“Yeah from the beginning we have gotten more and more tasks that they have given us to do. I feel very … I feel like safe with the things they have us do. They have been teaching and I can always ask like, 'What do you think, should I help the baby this way or not?' … so they … they really help a lot” (F5).*

Care activities such as lifting their infant to kangaroo care independently or preparing the milk to put in their infant’s nasogastric tube were described as tasks that families “really waited for” (F6) and were excited about.

Conversely, U.S. parents did not feel empowered by nursing support, and even suggested that it would be wrong for parents to expect to engage in their infants’ cares to the same level as the nurses did. One participant stated: *“When a baby is that medically fragile, there’s a lot of things you cannot do” (US2).* When opportunities to engage in infant care were allowed, they were often set on a strict time schedule that the parents had to adhere to. One participant describes missing her only opportunity to change her infant’s diaper that day due to traffic:

*“One time, I was driving from work down to the hospital, which is about a 35 minute drive, and it was traffic and bad weather, so the nurse went ahead and like changed him before I got there. I had called ahead and let her know I was running a little bit late umm, and she insisted that she changed him, ummm, because it was his care time and umm, she had to change him, and it, I mean it really upset me because there’s, there’s limited opportunities there to interact with your child” (US5).*

This approach to care spilled over into lactation. Participants in the U.S. felt that pumping was the only way they could provide for their infant because they were allowed few opportunities to care for their infant in other ways. This often created undue pressure to succeed with pumping. One participant stated:

“*And I would get so angry with myself. And I just, I mean I would pump every two hours for 20 minutes like the first probably month and it finally started working but I was very hard on myself about that, which is obviously due to the NICU because prior to having him I was like, ‘Yeah I’ll try it and if it doesn’t work who cares?’” (US6).*

Many also described the ways that pumping and adhering to the necessary pumping schedule felt like a chore because it did not come with the same relational benefits as breastfeeding: *“Having to set my alarm to pump in the middle of the night and not having a baby there was really, really difficult. I think having had that kind of difficult experience … umm, I also had to go to work … back to work, it took a toll” (US1).* When pumping was unsuccessful, many participants blamed themselves and internalized guilt at having to give their infant a lesser form of nutrition despite the uncontrollable barriers they had faced. One participant describes her emotions when she lost her supply: *“I was trying all of their tips and tricks but it just started to slowly wean off … I tried for a couple weeks to get it back up, and it was just, it wasn’t … it wasn’t what it had been. I was getting minimal … I cried for days” (US5).*

Some of the Finnish participants also felt a responsibility to provide for their infants by expressing milk. One Finnish participant described feeling as if it were her only role at first: “*It felt that if I am participating at least with the milk, I did something. Yeah it felt that I have, I have uh … a role. I have something” (F5).* While this is the case, many described the nurses’ efforts to remind families that they shouldn’t stress about lactation: *“The nurses reminded all the time that, that uh, you really shouldn’t feel bad if there isn’t enough milk … They would say it shouldn’t be a thing that you stress about, but uhh, also that all the benefits, that were really good” (F2).* These reminders, along with eventually gaining opportunities to independently care for their infants, offset any sense of failure in the Finnish participants. One participant stated:

“*On some days when I haven’t been able to produce as much milk, it feels … it feels like rubbish. Because that has been for a long time … the only think you can do for him. And of course, now it’s a lot more because I can do all the … uh be with him in other ways and kind of help him grow and get stronger” (F2).*

Outside of having a more healthy approach to lactation because of the additional ways Finnish participants were able to care for their infants, many Finnish participants also discussed how they were encouraged to take care of both themselves and their infants while in the NICU. One family described deciding to stay overnight in the unit only a few nights a week so they could rest, even if they wanted to always be in the unit with their baby. One participant stated:

*“Uh well when we stay here, we of course have been listening to all these sounds and it’s, it’s really difficult to sleep. We expect that we don’t get enough sleep … that’s why we take Sundays free. And so, so we want to experience it with her here, and then we just need to go home and rest for a bit” (F3).*

Others described finding time everyday to leave the hospital, go for a walk outside, or meet up with friends: “*It’s a bit like our home. It’s great … but everyday we try to do something outside or go for a walk... like go eat … yeah so … I have been meeting some friends too and stuff just to do something else. Everyday there is also something happening outside” (F5).* A similar sense of self-care was not present in the U.S. interviews, as many families described simply trying to stay afloat. One parent stated, *“I slept maybe four hours at a time, and I mean I made so many friends with all the nurses, I got teased because I would walk the halls, especially during the first couple of weeks when she was super fragile” (US5).* It is evident that when U.S. families were not provided the autonomy to act as normally as possible with their infants in the NICU, their own well-being was put at risk. However, when nursing support focused on the well-being of the entire family, participants in the Finnish unit were better able to care for themselves and in turn, care for their infants.

### Controlled or empowering breastfeeding experiences

Participants in both groups (seven in Finland; three in U.S.) were able to experience some form of breastfeeding in the NICU, whether this be non-nutritive sucking, simply “practicing” breastfeeding, or fully transitioning to breast. While this is the case, all participants had voiced a desire to breastfeed before they gave birth preterm and entered the NICU with breastfeeding as their goal.

For most U.S. participants, the inability to breastfeed was related to losing their supply before the infant was able to receive oral feeds or attempt breastfeeding. One participant stated: *“We were not allowed to do breastfeeding until they were on nasal cannula or off, off of the thing completely” (US4).* Often families believed that the extra support necessary to maintain their supply and make it long enough to attempt breastfeeding came too late:*“Um, yes, I had planned on breas- breastfeeding ... my kids. Um, once we met with the lactation specialist after I was discharged ... So they did go over all that, um, but only once I was out” (US7).* In addition to the insufficient lactation support and restrictive policies about breastfeeding initiation, it is also evident that breastfeeding opportunities were withheld even when the infant was indicating a readiness to feed at breast. One participant described losing the opportunity to breastfeed because her infant latched too well: “*We were able to do non-nutritive at first but she latched really well and really easily, so they didn’t want her to … to like overfeed … since they had to regulate every little bit that came in. Because she latched well, she never got to breastfeed” (US1).*

For those U.S. participants who were allowed to attempt breastfeeding in any form, this process was often slow-moving and stressful. One participant stated:

*“It literally would be like a drop, and that would exhaust him because his lungs, like, were so tiny and everything was tiny. So for me, the whole breastfeeding process was extremely long and drawn out, and numerous times I... kind of just wanted to give up, but I knew I wanted him to have my milk” (US4).*

It was clear to these participants that very few mothers in the unit were able to become successful with breastfeeding, which made them feel proud, but also limited their privacy to explore breastfeeding independently. One participant stated: *“I guess I know statistically from what I was advised there’s not a lot of moms that are able to breastfeed, whether the stress or they just aren’t able to produce milk, or umm... I know that there were literally a handful” (US2).* Some mothers described the attention their breastfeeding status garnered as a bit “awkward” even if the staff was well intentioned in supporting them. One participant stated:

“*Breastfeeding your child who hasn’t even been home yet in front of a nurse and … occupational therapy there as well. I think everyone just wanted to … I think because of them being on oxygen support, like lactation wanted to make sure that I was doing everything I needed to be doing, and OT was watching to make sure the baby was not shriveling or … ya know was in the best position to … to make it successful. So … it was very awkward as a new parent breastfeeding” (US4).*

For this reason, breastfeeding in the U.S. unit often felt sterile and controlled, making it difficult for mothers to use breastfeeding as a method for developing a relationship with their infant.

Unlike the U.S. participants, the participants in Finland described breastfeeding in the NICU to be a generally positive experience. In the Finnish unit, opportunities to practice breastfeeding were welcomed and usually allowed before the infant was off of respiratory support, including intubated infants on ventilation, as well as respiratory support via high flow nasal cannula and CPAP. Additionally, a restriction on total nutritional volumes did not prevent mothers from being able to attempt breastfeeding. Participants often described feeling overjoyed by their first time taking their infant to breast:

*“Well, of course it was really, well just fantastic...*. *And even the first times that, well, not even breastfeeding, but when they said that you could like bring him next to your breast, and kind of like smell, and maybe lick a little bit. So that was for me kind of the experience” (F2).*

Participants felt that taking their infant to breast was a critical part of building a relational bond even if the infant was not getting any milk from the breast. One participant stated, *“I think that breastfeeding is an important way to get close with the babies. And that it’s not just to eat, but to feel connected” (F5).* Another participant described feeling that her infant enjoyed being at breast regardless of feeding success: *“...and umm, and I feel like she was really enjoying it even though she didn’t get much out of it yet” (F3).*

The sentiment that breastfeeding was a dual responsibility and a dual benefit for both mother and infant was commonly discussed by Finnish participants. Some were surprised with the ways that their infants immediately knew what to do at breast, while others discussed the fact that they were both continuing to learn. One mother stated, *“Uhh it was very lovely to see that she right away got the point of what to do” (F6).* Even when breastfeeding was not successful at first, participants were encouraged not to stress and think of it as a learning process: *“We kept practicing and we both improve [d] a lot” (F3).*

The NICU staff often reiterated that the volume of milk transferred via breastfeeding should not be the focus of the experience. One participant stated, *“I felt that they were really supportive and uh, tried to emphasize that on the first times it doesn’t really matter … yeah … how effective it seems” (F2).* Once breastfeeding opportunities were offered, the nurses taught parents how to do pre and post weights to track the volume of milk transferred, but encouraged families not to feel discouraged by the numbers. One mother described the variability in her infant’s ability to successfully eat from breast: *“So the best that she can do at the moment is like 20, 20 mls [and] that’s actually quite a lot for a baby her age. So … yeah, but yeah it varies. Sometimes she can’t get much and sometimes she loses when I try to breastfeed her*” (F3).

Similar to the breastfeeding process in the U.S., Finnish participants also felt that the journey toward breastfeeding was slow moving and hard work. One participant stated:

*I would really love to fully breastfeed her, but it’s going to be a challenge I think that it will probably be worth it that we take the effort and try to progress till … till [full] breastfeeding so that she rarely eats from the bottle” (F6).*

While many Finnish participants hoped they would be able to transition their infant to fully breastfed, they were open minded to the idea that they may also have to give bottles: “*[I’m] trying to uh, let go of the thought that breastfeeding is the way (laughing) and that if it, if it feels too difficult it’s okay” (F2).* Overall, the experience of breastfeeding in the Finnish unit was realistic, yet positive and focused on promoting parent-infant closeness rather than focusing exclusively on nutritional value and milk volumes.

## Discussion

Our data revealed that care culture, beginning from the early postnatal period and continuing throughout the entire NICU stay, serves to frame the utilization and intended goal of lactation as relationally focused or exclusively nutritionally-driven. The primary finding of our study is that lactation was framed and utilized in each care culture to achieve a different end. The approach to lactation in the Finnish unit is grounded in finding methods to promote parent-infant closeness by focusing on the relational potential of breastfeeding. The approach to lactation in the U.S. unit is based in medical need for human milk and suggests that pumping is the primary method for parents to contribute to the health of their infant.

Previous studies have documented the varied experiences of mothers who are pumping for their infants in the NICU, and have also argued for a more relational approach to lactation using methods that promote closeness via pumping and breastfeeding [21, 57, 58]. This study expands these findings by showing that relational feeding has been implemented successfully in some NICU contexts, but not in others. The Finnish participants in our study seemed to experience “reciprocal” or “attuned” feeding with their infants [22, 59], and breastfeeding practice became a shared learning experience for both the mother and the infant. In contrast, U.S. participants felt singular pressure to maintain milk volumes and only experienced breastfeeding as controlled and medicalized. For this reason, promoting a sense of dual responsibility for breastfeeding while emphasizing relationship building may lessen or prevent the sense of failure common among mothers who are providing milk for their infants in the NICU.

While this study found many differences between the two care cultures, parental psychosocial challenges during the early postnatal period were common for both sets of participants. This finding is consistent with previous literature that regards the first few weeks after birth as the most critical for promoting parent-infant closeness and familial psychosocial well-being [60, 61]. This time period also coincides with lactation initiation. The “golden hour” of lactation continues to be clinically relevant due to evidence that early lactation initiation impacts milk volumes and breastfeeding status at discharge [62–64]. For this reason, neonatal clinicians should take into account the family’s ongoing adjustment to parenthood that is also occurring at this time and approach lactation sensitively. Prenatal lactation education for those at risk of preterm birth could help prepare parents to initiate lactation soon after birth. Furthermore, lactation education using a full family approach could reduce stress on the mother while redistributing the responsibility of lactation across the family unit.

Findings also showed that both culture and space played a role in the participants' overall NICU and infant feeding experiences. A feeling of “at homeness” in NICU spaces has been shown to relate to familial autonomy and a sense of attuned feeding [35]. Our study builds on this finding by suggesting that parents can better care for themselves and their infants in home-like spaces, but may feel a sense of failure or lack of control when they are simply visitors to the NICU. We argue that this is due to both space and nursing culture, and suggest that the Finnish participants’ increased involvement and healthier approach to lactation could be explained by the longer nurse-parent interaction known to occur in single-family rooms [34]. The symbiotic relationship between care culture and space is evidence that access to single-family rooms is not enough to encourage a relational approach to lactation. As this style of NICU architecture continues to become more prevalent, NICU staff members must be willing to alter their care practices to provide for the familial autonomy that these spaces encourage.

Our findings suggest that a focus *only* on the health benefits of lactation could be detrimental to family well-being. A true family centered approach is one that promotes family autonomy, parent-infant closeness, and shared-decision making [6], all of which may come into conflict with lactation when it is singularly framed as an end for health. Parents of preterm infants are already at an increased risk of mental health difficulties, which can have long-term impacts on neurodevelopmental and behavioral outcomes of preterm born children [65–67]. Therefore, strategies that promote family well-being should be at the forefront of neonatal research. This should include longitudinal research on the benefits of relationally focused lactation support for the mental health outcomes of both the infant and parents. We recommend that clinicians use a salutogenic, or health promoting, approach to all care, including infant nutrition and lactation promotion practices that protect breastfeeding. Relationally focused lactation will fall into place when the health and well-being of the family unit is at the center of NICU care.

### Strengths and limitations

This study builds upon the small body of literature on cross-national qualitative studies in the NICU and studies two cultural contexts that, to the authors’ knowledge, have not yet been compared, but represent varied approaches to FCC in developed NICU settings. Furthermore, with the ample attention that Nordic healthcare settings have garnered over the past decade, this study expands upon some of the potential reasons for their successes and recommends methods for improvement elsewhere.

This research was conducted at different points in time in both the development of neonatal care and in each participant’s NICU stay. Between the time that U.S. recruitment ended and Finnish recruitment began, care approaches in the U.S. might have changed to become more family-friendly. Additionally, because the U.S. interviews were conducted after hospital discharge, and the Finnish interviews were conducted in the last month of hospitalization, there could be some potential biases in both sets of interviews.

All of the interviews were conducted by S.H., who is a citizen of the U.S. and does not speak Finnish. In order to enhance credibility and promote reflexivity during analysis, research findings were grounded in field notes from participant observation in each setting and discussed regularly with C.F. and L.L. While all Finnish participants were proficient in English and interviews were conducted in English, the minimal language barrier present between S.H. and participants in Finland may have also introduced some bias into the interviews, even with the help of an interpreter, but the authors attempted to minimize this effect by having the interpreter confirm the quality and completeness of the transcripts.

There are minor differences between the study hospitals that may have influenced the participants’ narratives. While the infants of the U.S. participants were transferred to the children’s hospital after birth, this does not represent all NICU admission experiences in the U.S., as some NICUs are co-located with delivery wards. Hospital transfer may have impacted initial parental presence and lactation initiation for these participants, and must be considered when interpreting the results of this study.

Finally, the authors would be remiss not to note the differences in socioeconomic and ethnic demographics, family leave policies, and healthcare access between these two nations. The authors acknowledge that the general U.S. population is more socioeconomically and ethnically diverse than the general Finnish population, even if our sample populations are comparable, and that these attributes may influence care within cultural contexts differently. The authors also acknowledge the importance of generous paid family leave and universal healthcare in supporting FCC. These differences likely have an unavoidable impact on parental presence and care practices in both settings, and therefore must be acknowledged when interpreting the results of this study and making recommendations for FCC. However, despite these limitations, the findings of this study offer valuable information about ways NICUs can support the overall wellbeing of families, while raising important questions for future study regarding the role of societal structures in family NICU experiences.

## Conclusion

This study illustrates the importance of care culture to the infant feeding experiences of families, and suggests that it is both culture and space that should be accounted for when creating family-inclusive NICU environments. A global theme of *lactation as a “means” or an “end”* captures how lactation has been framed differently in these two unique neonatal settings. This finding suggests that relationally focused lactation that prioritizes family well-being can only be achieved by supporting parent-infant closeness and parental autonomy. Neonatal clinicians and scholars should take this into account when developing FCC initiatives and breastfeeding protective lactation interventions in the future.

## Data Availability

The data that support the findings of this study are available on request from the corresponding author. The data are not publicly available due to them containing information that could compromise research participant privacy/consent.

## Abbreviations

FCC: Family Centered Care
NICU: Neonatal Intensive Care Unit
VLBW: Very-low-birth-weight
